# Reviews and Book Notices

**Published:** 1879-01

**Authors:** 


### Reviews and Book Notices.

---

ARTICLE XV.—FIBROUS TUMORS OF THE UTERUS. A Clinical Lecture by W. H. Byford, M.D. (Seguin's Series.)

One interested in the progress of gynæcology must open eagerly pages which unfold the recent views of an expert respecting a field so fertile in doubts and discouragements heretofore, but now happily exhibiting some of its most brilliant successes.

Nor can there be any disappointment in store for the reader of the pamphlet bearing the above title, when he contrasts the usually limited scope of a clinical lecture with the fulness of diagnosis, adequacy of description, and the details of treatment here made manifest.

Dr. Byford, after examining his text cases, states that the relative frequency of these tumors, compared with those of ovarian origin has been, in his own experience, as ten to one; that they are homologous growths, but vary in regard to the ratio of their different constituent elements; in some ("myoma") the fibrous, in others ("fibroma") the connective tissues predominating, and that they originate in great and long-continued hyperæmia of the uterus.

Classifying fibroids, as usual, into submucous, intramural, and subserous, he clearly distinguished the characteristics imparted to them by reason of position and surroundings, and as well defines their retroactive influence upon the uterus itself. He accounts for their low degree of vitality and slow growth by the small size of their nutrient vessels, penetrating a condensed but not independent capsule, and shows how this fact contributes to a limitation of their development, and to their degeneration into lower forms of tissue, or to inflammatory disintegration. It is interesting here to read that "a single tumor grows more rapidly, causes more vascularity in, and greater hypertrophy of the muscular fibres of the uterus than the multinuclear form. The latter often reaches a limit for want of vascular supply, or is transformed into dense tissue of a vitality far below that of a single tumor. It sometimes becomes a true fibroid degeneration of the whole uterus, in which one would hardly trace any of its own anatomical elements."

"Fibroids seldom take their origin in the cervical tissues."

Symptoms will vary with the variety of tumor, hæmorrhage and menorrhagia being most common to the submucous form. Leucorrhœa follows the same rule as do watery discharges, and this because here the vascularity of the womb is greatest.

"Dysmenorrhœa, if present, is of the obstructive kind, and is most aggravated where the tumor is multinuclear and submucous."

Not to quote too largely, it is pleasant to find the author announcing a generally favorable prognosis, "because these tumors occur mostly in persons who approach the menopausis, and often cease growth afterward; they grow slowly and for years without causing dangerous symptoms, and often cease enlarging from no discoverable cause; they sometimes degenerate into inert masses, not particularly inconvenient, and nature will often dispose ot them by spontaneous expulsion; a judicious medical treatment is often efficient, or surgery interposes successfully for their removal."

In regard to the correlation of pregnancy and fibroid tumors, the author decides the former to be "most infrequent when the latter are submucous and confined to the central or upper zones of the uterus, and that the subserous forms are not so antagonistic."

In general, the larger the tumor the less likelihood of pregnancy, but if it occur, an impossibility of normal uterine development will lead to abortion. The danger of hæmorrhage under these circumstances Dr. Byford thinks to be over-rated.

The effect of pregnancy upon some of these tumors will be *nil*, but in very many cases, the subsequent process of involution set up in the uterus seems to extend to the morbid tissue and its decadence is thus assured.

Among remedies preventive of hæmorrhage, the author is particular to describe his method of preparing vaginal tampons, to be always on hand, and ready for introduction by the patient or her attendants. They consist of sponges, fine and large enough to fill the vagina, which have been saturated with a half strength dilution of the *liquor ferri persulphatis*, wound with a string to a proper shape and degree of compression, and laid away to dry. To introduce one of these when unwound, or to withdraw it by a cord passed through prior to suturation, will be no difficult matter. It may remain, if successful, forty-eight hours, when the hæmorrhage will be unlikely to return.

Considering the question of permanent cure, Dr. Byford alludes kindly to the " sorbefacient " remedies upheld by Atlee and others, but states that his own success with them has not fulfilled expectation. But he adheres positively to former views of the efficacy of ergot, which he affirms will cure one-third of the cases, and favorably influence two-thirds. In a recent address to the Obstetrical Society of Edinburgh, Dr. Alexander Simpson substantiates this opinion. Dr. Byford prefers to administer Squibb's fluid extract in doses of 1.33 C. C., gradually increasing till 12 or 16 Gms. are given daily. The larger doses are not used unless to promote expulsion of the tumor, and they frequently produce very distressing pain, which continues notwithstanding the withdrawal of the medicine.

Even opiates will at times fail to subdue the throes of the uterus till expulsion is complete, but the author affirms this violence of action to have done no real mischief in the cases under his own hands.

The question of the danger of using ergot in the subserous variety of tumors, Dr. Byford disposes of by calling attention to the fact that, in this case, the contractions induced are away from the axis of the tumor, and tend to pediculate it, or diminish its nutrient supply.

Gangrene of the extremities, or serious inconvenience to the nerve centers, have never fallen within the province of the author to see ; nor does he seem to be alarmed by the degree of septicæmia caused during a slow disintegration and expulsion of the growth.

The use of electrolysis by Drs. Kimball and Cutter receives passing notice, but with a very qualified approval.

The mention of surgical treatment is confined to that appropriate to sub-mucous fibroids, and the author's first remark is that this treatment must be as simple as possible, compatible with thoroughness. Its dangers arise (1) from laceration or other damage to the uterus resulting in hæmorrhage or inflammation; (2) incomplete ablation, causing septicæmia; (3) shock, sometimes following protracted efforts at removal.

Dr. Byford enumerates ligation, torsion and amputation, and describes minutely the manner in which each is to be effected, giving a decided preference to torsion wherever applicable.

One misses here the credit that should be given Dr. Emmet for his plan of "removal by traction," relying upon the closely following uterine wall to pedunculate the mass as withdrawn and separated.

It is evident from his whole treatment of the subject, that Dr. Byford is no alarmist, and the student would do well to consider the experiences of other eminent authorities if he would rightly weigh the dangers arising from hæmorrhage, shock and septicæmia during the spontaneous, medicinal or surgical treatment of uterine fibroids.

H. W. J.

ARTICLE XVI.—A CLINICAL HISTORY OF THE MEDICAL AND SURGICAL DISEASES OF WOMEN. By Robert Barnes, M.D., London, Censor of the Royal College of Physicians; Lumleian Lecturer (1873); late Examiner in Obstetrics and the Diseases of Women at the University of London and the Royal College of Physicians and Surgeons; Obstetric Physician and Lecturer on Obstetrics and the Diseases of Women to St. George's Hospital. With one hundred and eighty-one illustrations. Second American, from the second and revised London edition. 8vo., pp. 784. Philadelphia: Henry C. Lea.

The first edition of this excellent work having been for some time exhausted, the author has leisurely revised it, condensing many parts and adding one new chapter, on the "Relations of Bladder and Bowel Disorders to the Diseases of Women." He has also increased the number of the illustrations by cuts which are not only new but, like a large proportion of the others, are original with Dr. Barnes.

This being the second edition of the work, an extended review is unnecessary, but there are a few points to which we would call attention.

We are pleased to note the pains the author takes in various parts of his work to caution his readers against becoming *mere* specialists. In the beginning of the second chapter he says: "There is nothing special in the mode of studying diseases of women." "The general or distant affections require to be investigated and treated; but it is not safe to overlook the organs that may be secondarily involved." "And there are disorders of this special system commencing in it, and in their turn acting upon and inducing disorders in distant organs or in the general system." In the fifth chapter he says: "Do not concentrate all attention upon this one region of the body. Remember that the fault may be in distant parts: that disease in other organs may complicate disease in the pelvic organs." "The great clinical rule should be: Interrogate all the functions; examine every organ. In this way only can we acquire a well founded confidence that important disease is not overlooked; in this way only can we rightly estimate the relations of symptoms to disease, and the reactions of disease upon distant organs, and frame a rational plan of treatment." In another place—"We thus see how dangerous it is to practice in the spirit of pure specialism; how absurd it is to map out the body and assign particular territories to particular classes of practitioners. It will be seen how intimately—how indissolubly—that part of medicine which takes for its basis the particular study of the generative system in women is linked with the disorders of the alimentary, vascular and nervous systems; that is, a pure specialty cannot exist. A more monstrous thing cannot be conceived."

Expressions like these indicate the broad judgment and liberal culture of the author, and make us feel inclined to accept his teachings as safe and judicious.

The work is well condensed, and we do not know a more reliable one upon the subject.

A few things about the book strike us unpleasantly. On page

375 the author says: "Radford, imbued with the teachings of this master (Dr. Blundell), encouraged Clay at the commencement of that career which may be said to have placed ovariotomy upon a secure foundation." "The operation was suggested by William Hunter; its practicability and the mode of performing it were taught by John Bell; it was first practiced, and that successfully, by an American, McDowell, a pupil of John Bell." How grudgingly he gives a little credit to Ephraim McDowell, yet implies that most of the honor belongs to his own countrymen!

The book appears in handsome dress—good paper, clear type, wide margins and excellent engravings. H. P. M.

Article XVII.—The Throat and its Diseases. Lennox Browne, F.R.C.S., Ed., Senior Surgeon to the Central London Throat and Ear Hospital; Surgeon and Aural Surgeon to the Royal Society of Musicians, etc. Henry C. Lea, Philadelphia, 1878. Jansen, McClurg & Co., Chicago.

This book is designed as a practical guide to the diagnosis and treatment of diseases of the fauces, larynx and nasal cavity.

The author's endeavor has been to give prominence to those facts which will aid the practitioner in making an accurate diagnosis, and selecting appropriate treatment; but he has omitted discussions of purely pathological interest, having, instead, simply referred the reader, for them, to other works.

In describing instruments and methods of treatment he mentions only those which he has found most efficient, in his experience of eleven years of practice, devoted mainly to diseases of the throat.

He has given especial attention to the illustrations—about one hundred and fifty in number—one hundred of which are colored, so as to represent, nearly as possible, the condition in the patients from whom they were taken. These add greatly to the value of the book for those who have only a limited opportunity to study diseases of the larynx.

The first eighty-six pages are devoted to the description of laryngoscopic and rhinoscopic instruments, and the modes of using them; together with a description of the anatomy of the larynx and the semiology and general therapeutics of throat diseases.

Four chapters are devoted to diseases of the fauces, pharynx and nasal cavity, and the remainder of the book, six chapters, to diseases of the larynx.

The treatment suggested is not materially different from that recommended in other works of the kind, excepting in regard to morbid growths.

A single chapter treats of benign neoplasms in a light which will surprise many who have read of the brilliant results obtained by Lewin, Czermak, Morrell, Mackenzie, and others, by operative measures.

The author urges against instrumental procedures—that the functional disturbances produced by these growths is not usually grave ; that many of the growths will disappear under local applications ; that recurrence of the growths after removal is not infrequent ; that the instruments now employed are more dangerous than formerly, and that injury of the healthy parts may lead to fatal results, while the irritation incident to efforts at removal may cause a benign growth to take on a malignant character.

Though we are not prepared to give up operative procedures within the larynx, we have no doubt that many of the author's objections are valid, and they should deter those from operating who have not special experience in the treatment of diseases of the larynx.

This is a valuable work, and we take special pleasure in recommending it to the profession for its excellent illustrations, but especially on account of the letter press, which has been admirably executed in large clear type.

We commend this book as a model for all who wish to publish medical works, which are far too often rendered almost valueless by being printed with small or indistinct type, which renders their perusal painful, and by a prolixity which exhausts the reader's patience.

E. F. I.

ARTICLE XVIII.—A MANUAL OF PRESCRIPTION WRITING. By Matthew D. Mann, A.M., M.D., Lecturer on Clinical Microscopy, and Examiner on Materia Medica, in the College of Physicians and Surgeons, New York; etc., etc. New York: G. P. Putnam's Sons, 1878. Chicago: Jansen, McClurg & Co. 12mo. pp. 155. Price 90c.

The appearance of another work on prescription writing, so soon after the book of Gerrish, is a sure indication that the necessity of greater care and better knowledge in the matter of prescribing is being duly appreciated by the profession. The book before us is more extended than Gerrish's, without being unnecessarily elaborate in details.

Besides the usual directions for writing simple and compound prescriptions, information about weights and measures, rules for the grammatical construction of Latin prescriptions, forms and examples of extemporaneous prescriptions, it contains a complete list of officinal preparations, and a very valuable table of the doses of all substances in the primary list of the U. S. Pharmacopœia. The doses are given in both the old and metric systems. The introduction of a chapter on the metric system is certainly demanded by the times. The method of writing this system adopted in this book, is that advocated by the JOURNAL AND EXAMINER, and found in every foreign medical literature except the English.

F. C. H.

ARTICLE XIX.—REST AND PAIN: A Course of Lectures on the Influence of Mechanical and Physiological Rest in the Treatment of Accidents and Surgical Diseases, and the Diagnostic Value of Pain. Delivered at the Royal College of Surgeons of England, in the years 1860, 1861 and 1862. By John Hilton, F.R.S., F.R.C.S., etc. Edited by W. H. A. Jacobson, F.R.C.S., Assistant Surgeon to Guy's Hospital. Second edition. New York: William Wood & Co. 8vo., pp. 299.

The well-known publishing firm of William Wood & Co., of New York, have entered upon a new scheme to supply the medical profession with standard works at marvelously low prices. They have opened subscriptions for "Wood's Library of

Standard Medical Authors," which shall contain only works of recognized worth. Each subscriber will receive one volume each month throughout the year. Subscriptions are taken: 1st, The $12.00, payable on delivery of the first volume, in which case the volumes are all delivered free, by mail, from New York. 2d, Payable $6.00 semi-annually, in January and July, in which case the subscriber pays express charges on the January and on the July volumes, the other volumes being sent free, by mail; and 3d, Payable monthly at $1.25 per volume; in this case the volumes are each delivered free, by express or carrier, C. O. D.

The scheme commends itself to all physicians who are desirous of obtaining a library of valuable medical books for a small outlay of money. And the selection of such books as Hilton's work on "Rest and Pain," which is to be the January volume of "Wood's Library," shows that the publishers are in earnest when they promise to furnish standard works to their patrons.

We hope many physicians will avail themselves of this rare opportunity to fill their shelves with useful books.

ARTICLE XX.—HARVEY AND HIS DISCOVERY. By J. M. DaCosta, M.D., Professor of the Practice of Medicine at the Jefferson Medical College. Philadelphia: J. B. Lippincott & Co. Chicago: Jansen, McClurg & Co. Octavo pp. 57; price 75c.

This little volume consists of an address delivered at the opening of the session at the Jefferson Medical College. It is a very fitting tribute to the memory of this famous man, in the tercentennial year of his birth, and is interesting reading for every physician and friend of history of medicine.

